# Obstetrics, and Diseases of Women and Children

**Published:** 1860-03

**Authors:** Wm. B. Atkinson

**Affiliations:** Lecturer on Obstetrics and Diseases of Women and Children


### OBSTETRICS, AND DISEASES OF WOMEN AND CHILDREN.

BY WM. B. ATKINSON, M.D.,
LECTURER ON OBSTETRICS AND DISEASES OF WOMEN AND CHILDREN.

### I.—*Post-Partum Hemorrhage: its Prevention.* By T. GAILLARD THOMAS, M.D., of New York.

The frequency of this form of hemorrhage is, in a great degree, due to a neglect of the proper measures for its prevention. Contraction of the uterus, and that alone, will check any hemorrhage from its cavity. The causes of this accident of labors are inertia uteri, uterine exhaustion, sudden emptying of the womb, adherent placenta, accumulation of blood in the uterus, mental emotion, and an excited state of the circulation. The means for the prevention of hemorrhage are the observance of the following rules: After delivery of the head, permit the uterus to expel the body, unless it be absolutely necessary to interfere.

Let the hand of an assistant firmly compress the uterus, and follow the child down in its birth.

After the delivery, let the uterus be constantly held in the hand until the bandage is applied. Never apply the bandage until the delivery of the placenta. If the placenta is detached, remove it within twenty minutes. Avoid stimulants during and after delivery.

Remain in the *lying-in chamber* for one hour after delivery. Before leaving the house always apply the child to the breast. Enjoin perfect quiet and exclusion of visitors.

If there is reason to anticipate hemorrhage, give ergot as the head is passing the perineum.

He analyzes each rule, and gives his reasons for it in detail.

He objects to the application of the bandage prior to the delivery of the placenta, because it becomes saturated with the blood which has collected, and that which accompanies the expulsion of the placenta; it prevents the observance of the second and third rules, and the recognition of the dilatation of the uterus; its application annoys the patient when she most needs rest, and possesses little value to prevent harm. In the wards of Paul Dubois, at Paris, the bandage is never used, and yet hemorrhage is as rare there as elsewhere. Stimulants should only be used when required by syncope or extreme debility, as they increase the force of the heart's action, render the patient restless and uncomfortable, and produce an excited condition predisposing to hemorrhage. A large majority of the cases of hemorrhage occur within the first hour, and especially the first half hour succeeding delivery; hence the propriety of remaining, as the physician can at once meet the impending danger when it is first commencing to be formidable.

The application of the child to the breast exerts a reflex influence upon the uterus of a most salutary kind. This has even been found to act by increasing pains which are tardy in labor ; it has been recommended by Scanzoni to apply a child to the nipples, or for the nurse to stroke or otherwise irritate them. Rigby and Marshall Hall equally agree upon the value of this method. The value of the other rules is too obvious to require extended remarks.—(*New York Journal of Medicine*, January, 1860.)

### II.—*Uterine Catheterism with Cat-gut, as a Means of Producing Artificial Premature Labor.* By DR. C. BRAUN.

In substituting the cat-gut for the uterine sound, and injections, M. Braun proposes especially to avoid those lesions of the membranes to which the employment of the other two methods so easily gives rise. The bougies which he uses are one foot in length, and two or three lines in diameter. Before introducing one of these into the uterus, it is softened at one end to the extent of a third of an inch, by being dipped in hot water. It is then anointed and passed into the uterine cavity, directed by the index finger of the left hand, and pressed in by a gentle rotation. It should be arrested after having passed about twice the breadth of the finger into the orifice of the neck.

M. Braun prefers these bougies to gum-elastic probes ; he has always observed them to provoke the pains about the end of twenty-six hours. He withdraws the bougie a short time prior to the rupture of the pouch of waters or the birth of the infant. He has employed uterine catheterism, either with the cat-gut or with the elastic probe, twelve times during the years 1857 and 1858, for the purpose of producing premature labor ; of sixteen infants, he recorded eleven who lived, and five still-born. Eight mothers were saved, and the four who died succumbed to causes foreign to the operation, as pneumonia, tuberculosis, Bright's disease. The operation was performed five times with the cat-gut, and four times with French, very flexible, gum-elastic probes; in none of these cases were the membranes broken. This accident, on the contrary, he was not able to avoid in three cases where he employed probes which were very slightly flexible, obtained from English instrument makers.—(*Wiener Medizinische Wochenschrift*, 1858, No. 46 ; *Gazette Hebdomadaire*, December 2, 1859.)

### III.—*Retroversion of the Uterus in Pregnancy.* By R. BARNES, M.D., etc.

Retroversion of the gravid womb is a displacement by which the organ is dislocated from its normal erect, or slightly forward inclining attitude, and thrown back, revolving on its transverse axis, so that the fundus becomes impacted under the projecting promontory of the sacrum. In most cases the *cervix* and *os* are carried upward and forward behind the symphysis. There is more or less of retroflexion accompanying this affection. There are two distinct forms; in one it is produced gradually, in the other suddenly. Retroversion is exceedingly rare in women pregnant for the first time. In the gradual forms there exists some degree of prolapsus at the time of conception and afterwards. The uterus being thus low in the pelvis, grows in that situation, and on reaching a certain size, instead of rising out of the cavity, it projects itself against the promontory. Continuing to enlarge, it is gradually turned upon its transverse axis. When it fills the pelvis, the symptoms of pressure are developed. The obstruction of the bladder, formerly slight, is now constant. In the sudden form, it is produced in a different manner, although here too there must exist a predisposing condition. Under powerful straining efforts, the pressure of the abdominal muscles is thrown upon the *fundus uteri*, which is thus driven back under the promontory. Or, a woman encounters a violent concussion, and it is found that the womb has been thrown down within the pelvis.

The prominent symptoms are the great desire to empty the bladder; hence arise straining, tenesmus, and pains simulating those of labor. To these may be added uræmia, if the bladder is not relieved. The diagnosis may be made by examination, externally, of the abdomen, per vaginam, per rectum, and per vesicam. When the tip of the finger enters the vagina, it is arrested by a solid globular body, only permitting the finger to pass up with difficulty between it and the symphysis, where the *os uteri* will be found close behind, and above the level of the *crista pubis*. Per rectum, a large, solid, globular tumor will be found in the hollow of the sacrum, compressing the rectum. The examination per vesicam must be made with a male flexible catheter, directed well forward. When the bladder is empty, the abdominal tumor, which may at first have been mistaken for a gravid uterus or dropsy, has disappeared. The abdominal walls become flaccid, admit of free examination, and, on feeling above the pubes for the womb, that organ is not found. Hence the conclusion is that the tumor felt per vaginam, filling the pelvis, is the gravid uterus.

The treatment must vary according to the state of the case. The bladder must be emptied three or four times in the twenty-four hours. Unload the rectum by warm water enemata. Then, by making the woman lie in the prone position, with the pelvis raised, frequently spontaneous reposition will occur, and the cure be effected.

If more serious, manipulation must be employed. Never attempt to hook down the *cervix* by pressing the finger on the *os*. Introduce the whole hand into the vagina, doubling the fist, and apply the flat surface made by the first phalanges to the fundus, and thus make pressure. The fingers alone, thus applied, would be extremely liable to cause detachment of the placenta, should it lie upon that wall of the womb thus pressed and indented. At the same time, the patient should be placed on her elbows and knees to obtain the aid of gravitation. Reasonable force *only* must be employed. If the attempt fails, give an opiate, and let her rest. Next time employ chloroform, and place the patient on her left side, in the usual obstetric position. Should reduction still be impracticable, and the symptoms be urgent, lessen the bulk of the distending body, by producing abortion. The liquor amnii may be evacuated by a catheter, or a douche bath applied. Finally, puncture the walls of the uterus. If this is attempted by the vagina, the trocar must be directed perpendicularly to the uterine walls, hence point it obliquely backward to the hollow of the sacrum. After the evacuation of the liquor amnii, as the danger is less urgent, the attempts to reduce the uterus may be suspended for awhile. Nature will expel the contents of the womb, and by involution its bulk will be lessened, and there will be a spontaneous termination of the difficulty. Opium and chloroform are the most useful means to relieve pain and spasms. Stimulants and nourishment must be given to support the strength, and uræmic poisoning may be met by nitromuriatic acid and ether.—(*Lancet*, December 3, 1859.)

### IV.—*Exhaustion after Suckling.*

Protracted suckling, especially when the supply of milk is scanty, is often followed by extreme debility and exhaustion, with the presence occasionally of various anomalous symptoms. A young woman aged twenty, the mother of two children, was treated at St. Bartholomew's Hospital for an attack of this kind, associated with diarrhœa. She gradually became much exhausted, had fits of shivering, with cold night-sweats. Some six weeks prior to admission, the diarrhœa set in, and remained persistent; it stopped two or three times and recurred, and thus assumed an intermittent character. She was ordered four ounces of wine and suitable nourishment, and an aromatic draught containing eight minims of tincture of opium. The diarrhœa was completely checked, and she began to gain flesh and strength. This is but one of many such instances, illustrating the folly of mothers in persisting to suckle their offspring when their strength will not permit. In other cases, dimness of vision comes on, and even temporary amaurosis has occurred.—(*Lancet*, December 3, 1859.)

### V.—*Two Cases of Closure of the Womb Successfully Treated.* By R. L. MACDONNELL, M.D., of Montreal.

*Case* 1. A married lady, who had suffered for some months with uneasiness in the lower part of the abdomen, dysuria, pain and fullness over the region of the ovaries, accompanied by a constant creamy discharge from the vagina; she had not menstruated for six months, and was fast losing flesh and strength. On examination, the abdomen was found to be soft, soreness was felt on pressure above the pubes, and over the inguinal region; evidences of inflammation of the cervix uteri extending to the body of that organ, together with acute vaginitis, were discovered. The sufferings were great during the acts of defecation and micturition. Large doses of morphine had been taken to relieve the intolerable agony. The treatment consisted of scarifications of the cervix and the application of argenti nitratis to the inflamed surface, followed by sedative and astringent lotions. These produced almost immediate relief, and though the narcotics were discontinued, she slept well from the first day. Within a fortnight she was quite well.

About six months after, similar symptoms arose, and from various causes she was unable to see Dr. MacDonnell for more than a year, and though treated by other physicians, with cauterizations, etc., no improvement was observed. When again able to place herself under his charge, she was thin, weak, very anæmic, had an excessive elevation of the right inguinal region, a well-developed ovarian enlargement, from whence shooting pains extended to the loins and thighs, having been compelled again to resort to large doses of morphia. On an examination by the speculum, the vagina presented a pale appearance, the cervix shortened and indurated; the os being closed by a dense, resisting membrane; with a diminution in the capacity of the vagina, and a contraction of all the structures. The uterus was not larger than natural. General treatment was employed, and counter-irritation over the inguinal region, under which much improvement was produced, with the exception of the distressing pains. In order to remove the obstructions of the os, he engaged the cervix in the speculum, ascertained a central spot which offered least resistance to a probe, painted cerate around it, and then applied potassa fusa by means of a sharp point of wood dipped in the caustic, rendered deliquescent by exposure to the air. Vinegar was then used to neutralize the escharotic, and in a few days a small slough fell out. The operation was repeated, and after a few days more a fine cat-gut bougie was introduced through the opening into the cavity of the womb. One of Guthrie's urethrotomes was passed in, and two incisions made, after which a No. 12 gum-elastic bougie was inserted daily, and allowed to remain for some hours. The general health was now completely restored, she became regular, and the cure was complete.

*Case* 2. A stout, plethoric female, aged thirty-five years. Had "closure of the womb" for nine years. Before this occurred, she was instrumentally delivered, after a tedious labor, of a dead child. She never menstruated afterwards. Six years after the delivery, the vagina was found to be closed, about half way between the orifice and the cervix, by a strong membrane, which was divided by a crucial incision. This gave exit to a small quantity of bloody fluid, which continued to flow for several days. She returned home, and after three years again consulted Dr. MacDonnell, who then, on examination, found the abdomen much enlarged, the vagina terminating in a cul-de-sac. She seemed in perfect health, yet daily suffered the most excruciating torments, which lasted for three or four hours. He proceeded to operate as before. Made a slough with the caustic, enlarged the opening by tents of gentian root, till a full-sized gum-elastic bougie could be introduced. Yet no fluid passed, nor were her sufferings relieved. A metallic catheter could only be passed an inch and a half. By further examination he found, as above, the cervix with the os closed by a dense structure. While the fundus uteri was pushed down by the left hand, he made some crucial incisions with a bistoury, and this not seeming to open the cavity, he passed up the index finger of the right hand, which, after some manœuvres, entered the cervix, and immediately a gush of a dark fluid, like treacle, took place. About half a gallon escaped, and the flow continued for two or three days. The abdominal tumor subsided, the pains were all gone, shortly the menses returned, and the cure was complete.—(*British-American Journal*, January, 1860.)

### VI.—*On Granulations of the Lining Membrane of the Uterus.* By Dr. James Trudeau, of New Orleans.

This affection, though frequent in the South, is comparatively rare in the Eastern States and the north of Europe. Uterine fungosities are true *fibro-*

*plasms,* located in the neck and cavity of the uterus, and are found in two forms.

1. Small tumors, with a wide base, springing directly from the mucous membrane; in size varying from that of a pin's head to that of a strawberry, the larger being often pediculated. The surface is rough and irregular, of a soft consistency, the growths being easily detached. The color varies from pale pink to red. This variety is generally found near the implantation of the Fallopian tubes, and is designated by the name of *cellulo-vascular vegetations.*

2. Pediculated vegetations, smaller than the preceding; of a grayish color, of a greater density, elastic, and smooth. Generally found in the neck and inferior segment of the uterus. These are not readily detached, and are the *cellulo-fibrous vegetations.*

According to M. Charles Robin, these vegetations consist of a hypertrophy of the mucous membrane, with numerous cells, fibro-plastic elements, and an increased vascularity for the first variety. The second has the same structure, with fewer vessels, and is protected by a layer of epithelium. They are both sometimes found together, and sometimes are observed at the orifice of the *os uteri,* bulging out of the canal.

The symptoms consist at first in an increased menstrual discharge, which soon becomes a profuse hemorrhage, succeeded by watery leucorrhœal effusions. The latter is sometimes very painful. Dysmenorrhœa occurs; the menstrual periods encroach on each other, causing the patient to be constantly flooding; the digestive functions are disordered, with the accompanying train of symptoms of dyspepsia; sharp pains are felt at the lumbar, sacral, and hypogastric regions; or there is a painful spot in one of the iliac regions, extending to the corresponding limb. The patient loses flesh rapidly; the features assume that peculiar cast of countenance observed in many uterine disorders (*facies uterina;*) the skin loses its softness and brilliancy; the eyes are sunk deeper in the orbit, and surrounded with a bluish circle; the extremities are always cold, even during the warmest weather. Examination by touch shows an enlarged uterus, and softness of its walls. The vaginal portion of the neck is often hypertrophied, and softer than usual; the canal much dilated. The speculum discloses an open *os,* discharging a thick, tenacious, transparent, yellowish-white mucus. The neck is congested, and dotted with minute superficial excoriations. The sound is easily introduced when the disease has extended to the cavity of the womb. Otherwise, much difficulty is experienced in passing it. When introduced, it can be moved in any direction, showing the increased capacity of the organ. The introduction often causes hemorrhage. There is no pathognomonic symptom, yet, with care, the differential diagnosis may be made. Uterine polypi may simulate this affection, yet an error of diagnosis in this respect would be of no importance, as the treatment is the same. In a case of simple menorrhagia, the hemorrhages, patulous condition of the os, and softness of the vaginal portion of the neck, may induce the belief that they are due to the presence of fungosities. In both dysuria and this affection there are severe lumbar and hypogastric pains. In menorrhagia the hemorrhages occur at the menstrual periods; in the other affection, they are almost continuous. The leucorrhœa is generally confined in the former to a few days before and after the periods; it is constant in the latter. In the former, the cavity of the neck is pale; in the latter, it is red and congested. The affection progresses slowly; the prognosis is generally favorable, and only otherwise from complications. M. Nélaton says this affection is observed in females who have passed the prime of life; seldom before the twenty-fifth year, and oftener at thirty-five. M. Richet confirms this, (which is in perfect concurrence with our own observations,) and remarks further, that it can always be traced to a confinement or miscarriage.—(*N. O. Med. and Surg. Jour.*, January, 1860.)

VII.—*Diagnosis of Ovarian Tumors.*—By Washington L. Atlee, M.D., of Philadelphia.

When disease of the ovaries is going on, slight evidences exist prior to its rise above the brim of the pelvis. After that, there is found a tumor, varying in size, of an oval form, and to one side of the median line. In examining, the patient should be on her back, and slightly covered. The tumor is circumscribed, elastic, movable, seldom sensitive. As it increases, it takes a more central position, and encroaches on the abdominal contents. If unilocular, fluctuation does not extend beyond the borders of the tumor. If multilocular, hard masses will be felt. Percussion gives a flat sound over the tumor, and a resonance at its borders. All these symptoms may be absent, or diametrically opposite, and yet ovarian disease be present. Usually, the general health is undisturbed. At other times there may be indigestion, and its accompanying troubles, diarrhœa, hemorrhoids, anasarca, dyspnœa, and great emaciation. The catamenia may go on naturally or may be absent, hence this point is of small moment. When the form of the abdomen is symmetrical, the cyst may be inferred to be unilocular; when elevations and depressions are observed, it is multilocular, or there are solid growths. The examination should be carefully repeated, with the patient lying on each side successively, as well as supine, and semi-recumbent. The evidence obtained should then be compared, and if the dullness is the same, the resonance and fluctuation do not vary, the tumor may be considered as ovarian. A rectal and vaginal examination are equally necessary. The uterus may be displaced in a variety of ways. There may be also prolapsus of the bladder and vagina. The womb-sound will enter the uterus about two and a half inches. The isolation of this organ is of value in regard to operating. Tapping is an important means of diagnosis, but should only be used to relieve urgent symptoms, or to decide the question of an operation, which should never he performed without it. In the only two operations in Philadelphia, where no tumor was found, previous tapping was omitted. A large trocar and canula should be employed; the lancet being highly dangerous, as, if adhesions do not exist, the fluids may be poured into the cavity of the peritoneum. The position should be on the back in bed, with the head and shoulders elevated. The point for inserting the trocar should be in the *linea alba*, about two to four inches below the umbilicus. It is better also to slit up the *cutis vera* with a bistoury, and then insert the trocar. Dry tapping can never occur when fluctuation has been previously ascertained. An unadherent unilocular cyst will sink as the contents flow out, and the resonance on percussion will be above where a dull sound was formerly heard. Tumors vary in composition; thus there may be a number of cysts, and as one is emptied, it sinks, and the trocar must be passed in to the others. A rare complication is a large ovarian sac, immersed in a great quantity of peritoneal fluid. On tapping, this escapes, and the existence of the tumor becomes evident. This being perforated, a different liquid is found. Sometimes the contents are viscid and will not flow out. In cases of colloid tumor, fluctuation may exist without any fluid. This is valuable negative testimony. The discharge must be examined, chemically and microscopically. It may resemble starch-water, coffee, dregs of urine, soapy water, etc. It may be green, inky, gelatiniform, purulent, etc., or contain crystals of cholesterine, tufts of hair, and other substances.

When a clear, transparent serum flows out, care should be taken, as this rarely comes from an ovarian cyst. If albumen is present, heat or nitric acid will produce coagulation. By the microscope, cell forms will be found in abundance, rich in oil, combined with blood globules, epithelial scales, pus, and pyoid forms. The peculiar cell is of a fine granular appearance. There is no other fluid corresponding in character, hence a fluid with these characteristics may be regarded as pathognomonic of ovarian disease, and in a large majority of cases will be perfectly reliable.—(*Philadelphia County Medical Society.*)

VIII.—*Ovarian Tumor Simulating Pregnancy.* BY W. C. B. FIFIELD, M.D., of Weymouth, Massachusetts.

The patient, aged twenty-four, mother of one child, in April perceiving a change in her health, felt certain she was pregnant. Vomiting came on, ceased for a fortnight, and again occurred, continuing irregularly for awhile, and *then continued day and night* from the last of July till the last of October. She was confined to bed most of the time; all food was rejected except molasses and water. Remedies best borne were calomel and effervescing draughts. The menses were regular till September. Occasional pains were felt in the back and abdomen for some months. She increased in size so rapidly, that by the sixth month she was much larger than a woman commonly is at the ninth. No motion was felt. Urine scanty. Dyspnœa not marked. In October she was much emaciated, there was no œdema, the tumefaction of the abdomen reached to the ensiform cartilage; fluctuation evident; the form of the belly was completely that of pregnancy. Directly below the pubes a hard body was felt. No trace of the uterus could be found, on examination by the rectum. A catheter was easily passed into the bladder; very little urine. No hardness or irregularity were to be felt in the abdomen. Upon auscultation with a Camman's stethoscope, no placental souffle was heard; but Dr. Fifield thought he could hear the fœtal heart at the right side. In opposition to its being simple ascites were the form of the belly, flattened at the sides; absence of œdema of the legs; the fluctuation, although present, gave the idea of the presence of but a small quantity of fluid; the umbilicus not being protruded or thinned; the finger, thrust suddenly into the umbilicus, came down upon a solid tumor like the fundus of the uterus; the absence of thirst.

Opposed to its being pregnancy were the state of the os and cervix uteri, absence of the placental souffle, and the absence of motion. In favor, were the form of the tumor, the impossibility of detecting the uterus by the rectum, the supposed sound of the fœtal heart, (the absence or presence of the placental souffle being no argument either way,) the vomiting, and the pains felt by the patient. The uterine sound of Simpson could not be passed beyond the os uteri, in an attempt to ascertain whether it might not be dropsy of the amnion. A Vallex's sound, thin and flat, was passed up to the *fundus uteri*, the organ being found completely anteverted, the sound lying almost horizontally, the handle very far back toward the rectum. The body beneath the pubes was the *fundus uteri*.

The patient was turned on her right side, and a large trocar passed through the linea alba, giving exit to a gummy fluid, amounting to about eight quarts. Masses like soft soap also passed out. Death occurred on the twelfth of November.—(*Boston Med. and Surg. Jour.*, December 1, 1859.)

### IX.—*Treatment of the Hysteric Paroxysm by Chloroform.* By M. BRIQUET.

The hysterical paroxysm should not be left to itself, inasmuch as it becomes a predisposing cause of future attacks, and always produces an injurious disturbance of the economy. M. Briquet arrests it by chloroform. A little dossil of charpie is wet with this fluid, and applied to the nostrils, the mouth being closed. In a short time the agitation ceases, and the attack is cut short. After a little time the patient awakes with a slight headache, perhaps the effect of the chloroform, and is soon as well as before. During the first few inspirations the agitation is considerable, and the patient requires to be held; this, however, soon passes off, and a tranquil sleep comes on. Hysterical patients seem to be very susceptible to the effects of this remedy. M. Briquet has used this agent thus for twelve years, yet has never seen either convulsions, coma, or dangerous syncope after it; the resistance is slight, if any, and the convulsive condition imparts to these patients a muscular power and an amount of vital resistance which entirely protects them from the accidents resulting from debility. The remedy succeeds in nine out of ten cases. Those who prove refractory are the robust, of sanguineous temperament, and in whom the attack is very violent. In such, the good results are temporary. Fortunately, they are rare. When the paroxysm seems to originate from a painful point in the limbs or trunk, which is quite common, the same agent applied topically during the intervals frequently causes the pain to disappear, and puts a stop to the attacks thus induced.—(*Med. Times and Gazette*, January, 1860; *Archives Gén.*, xiii., 664.)

### X.—*The Mouth to Mouth* vs. *The Marshall Hall Method in Asphyxia Neonatorum.* By A. T. KEYT, M.D., of Ohio.

The case of the asphyxiated new-born child is not quite parallel with that of the adult. The former has never respired; its chest and air-vessels not having been expanded, there is not that resiliency which is so important in the "Ready Method." The case, however, presents many characteristics rendering the old method the most suitable; from the size of the child, it is easily placed in any position; its small mouth can readily be covered by that of the physician, whose force is quite sufficient to expand its delicate lungs; the child is readily quickened into life by contact of the air with its air-vessels; and no difficulty is experienced by the falling back of its tongue. Then, too, under the old method, there are unmistakable signs that the indication is being met, the thorax expands, the air enters; the thorax contracts, the air escapes. A number of cases are related to show its advantages. The "Ready Method" also having been employed in the same cases, its negative qualities were apparent.

It is certainly true that the new-born child bears the deprivation of air with peculiar tolerance, and this is an incentive to persevere. No time, however, is to be lost, but the proper measures at once instituted. The "Ready Method" may act excellently after respiration has been once established, as a means of resuscitation. When compared together, it is evident that the old plan is as ready and easy as the new.—(*Cincinnati Lancet and Observer*, January, 1860.)

XI.—*Hemorrhage from the Bowel in Children as a Sign of Polypus of the Rectum.* By THOMAS BRYANT, Esq., F.R.C.S., Assistant Surgeon to Guy's Hospital.

The connection between hemorrhage from the bowel in children and the existence of a polypus of the rectum is so constant, that it is astonishing that it is not more generally recognized. In the majority of cases under Dr. Bryant's care, it had existed for months; all were under ten years of age, and many had been treated for piles. In some cases the discharge is constant, and in these the polypus will generally be found within, if not protruding from the sphincter. In others, the discharge is occasional, and generally accompanies defecation. There is generally straining after stool. Although the disease is troublesome and debilitating, yet it is easily treated, and rapidly cured. He reports a number of cases, in all of which recovery speedily followed, after the removal of the polypus by means of the forceps.

When hemorrhage is observed, the surgeon should at once examine the part, when the growth will most probably be easily detected. The structure of the tumor is simple, presenting the characters of a fibro-cellular growth. After removal, no treatment is required. Dr. Bryant believes that the cases so frequently reported of children suffering with piles are, in reality, instances of the disease now under consideration.—(*Lancet*, January, 1860.)

XII.—*Laryngismus Stridulus.* By A. JACOBI, M.D., of New York.

This is emphatically an infantile disease, and is observed in both healthy and sick, asleep or awake, playing, eating, or singing. The first stage is a sudden and entire apnœa. Respiration is stopped for a few seconds, or even a minute, the face is pale or bluish, the skin cool, the heart's action scarcely perceptible, the muscular system paralyzed. Reaction, or the second stage, is shown by a violent, deep, crowing inspiration. The third stage is that of complete recovery. In severe cases, there may also be tonic convulsions and involuntary evacuations. It is seldom fatal, and is apt to last for months. Death, when it occurs, is always seen in the first stage. These symptoms can only be explained by a functional trouble, a paralysis perhaps of the spine and nervous centres. Its duration is various, some having it but once, others for months or years. It is most frequent about the commencement of dentition. The mildness or severity of the attacks depends on the constitution and occasional causes. Hypertrophy of the thymus gland has no influence in the production of this disease, as was once thought; hence the name "thymic asthma" is incorrect. It may arise also from disturbance of the alimentary canal, by any cause, as excess in feeding, and dentition. It is highly probable that hypertrophy of the thyroid gland is a cause. The treatment has been very various. The indication is the thorough irritation of the respiratory muscles. Electricity would be of great value, could it be applied at the right moment. The patient should be kept in a sitting posture, allowed plenty of fresh air, the face sprinkled with cold water, and ice or cold water applied to the sternum. Artificial respiration, kept up till the paralysis has been removed, has proved of value. To prevent a return, attend to any cerebral disease. Tonics should be exhibited, if demanded; nutritious and digestible diet given; anti-scrofulous treatment, cod-liver oil, iron, and iodide of iron, etc. Much circumspection is needed in selecting the means of removing the disease. One case recently has shown the necessity of mental education. A little girl aged eleven months was affected so as to become "asphyctic" whenever contradicted. A pailful of cold water was ordered to be kept ready, and poured over her as soon as any symptoms of an approaching attack presented. In the course of a week, three or four such doses proved sufficient to soothe the temperament of the patient, and to entirely remove her attacks of laryngismus stridulus.—(*New York Journal of Medicine*, January, 1860.)

